# The Impact of Social Capital on Mental Health: Evidence from the China Family Panel Survey

**DOI:** 10.3390/ijerph19010190

**Published:** 2021-12-24

**Authors:** Xianhua Dai, Nian Gu

**Affiliations:** 1 School of Public Administration, Central China Normal University, Wuhan 430079, China; gn1684168437@163.com; 2 Center for Labor and Social Security Research, Central China Normal University, Wuhan 430079, China

**Keywords:** social capital, mental health, instrumental variable, two-stage least square, robustness, heterogeneity

## Abstract

The influence of social capital on mental health is a controversial topic. As some studies have pointed out, cognitive social capital significantly affects mental health but structural social capital does not. Using data from the China Family Panel Survey, this study measured social capital from social help, social trust, social networks, and social participation, and took regional average level of social capital as the instrumental variables, and applied a two-stage least squares regression. We found that the mental health of residents who trust and help each other is significantly higher than that of residents without trust and mutual help. When residents’ efforts to maintain social networks increase, their mental health significantly improves. These results are robust. Furthermore, the impact of social capital on mental health was heterogeneous in terms of urbanicity, gender, age, and area. These results are helpful for making policies for promoting residents’ mental health.

## 1. Introduction

With the development of society, people have paid more and more attention to mental health. As a substantial body of evidence has documented, mental health is affected by factors such as the environment [1], living habits [2], medical resources [3], and experience [4]. It relies on economic development and community policies to improve mental health by increasing individual income and providing a better living environment, advocating healthy lifestyles, and promoting the rational flow of medical resources [5,6,7,8,9]. However, even though the state and society promote mental health through a variety of ways, mental illness is still severe [10]. As the China Mental Health Survey indicated, the number of Chinese people who suffer from depression reached 95 million by 2015 [11]. Thus, it is necessary to expand research on the factors influencing mental health, with a focus on social capital [12].

Originating from the field of sociology, social capital has been a great concern in public health since the 1990’s [13]; however, there is still a lack of consensus on the definition of social capital [14]. Social capital may be the resources mobilized by individuals or collectives to realize their interests [15,16]. Social capital can be also viewed as the features of family, peers, the community, school, and work [17,18,19,20]. According to the functional perspective, social capital is divided into outreach and cohesion [21]. In addition, the standard content of social capital can be divided into cognitive social capital and structural social capital [22,23,24]. Although there is no consensus on the concept and classification of social capital, there are common elements, such as formal and informal relationships, mutual assistance, trust, and social participation [17,25].

Although a lot of research has been carried out, the impact of social capital on mental health is a controversial topic. A meta-analysis showed a very small impact of social capital on mental health [26]; some studies also support these findings [22,27]. However, there is also a lot of work indicating that social capital has a significant impact on mental health [28,29,30]. Some of the literature has demonstrated the impact of social capital on the mental health of special groups, such as adolescents [31,32,33], ethnic minorities [34,35], immigrants [36,37], the aged [38,39], and the diseased population [14,40]. In addition, as the research on the specific sub dimensions of social capital has documented, cognitive social capital, cohesive social capital, and community social capital have significant impacts on residents’ mental health [21,41,42]. However, overall, most of the literature does not address the causal inference. Minority literature on the paths has indicated that social capital leads to health inequality through the social and economic inequality of individuals and families, health investment, and the interaction between individuals and their environment [43,44]. In addition, social capital may affect an individual’s mental health by influencing their attitude to life and habits [24].

There are many studies accumulated so far on the impact of social capital on mental health, but most of them do not address causal inference. This study explores the causal inference on the impact of social capital on mental health in China. The remainder of this paper is structured as follows. Section 2 contains the data, definitions, and a summary of the variables. Section 3 outlines the empirical approach. Section 4 presents the results of the two-stage least square (TSLS) regression, a robustness check, and a heterogeneity analysis. Section 5 provides a discussion. Section 6 concludes.

## 2. Data and Variables

The data used in this study come from the China Family Panel Studies (CFPS) conducted by the China Social Science Investigation Center at Peking University. CFPS is an ongoing longitudinal survey which started in 2010. The data are collected in interviews once every 2 years. The study is based on a permanent sample of 14,960 households and 42,590 individuals who entered information in the 2010 baseline survey. It conducts a detailed and comprehensive investigation of family income, social security participation, education, health, and individual characteristics, which makes these data suitable for the research and analysis presented here. Additionally, it has a large sample size, wide coverage, a reasonable questionnaire design, scientific survey methods, and timely data updates, effectively reflecting the development of our society.

In this study, we focused on the relationship between social capital and the mental health of adult individuals. We started with 32,669 respondents from the CFPS samples in 2018 and applied some sample restrictions. First, we deleted the respondents who were still in school at the time of the survey (4075). Secondly, in the CFPS, individuals over 17 years old are defined as adults, so we deleted samples aged 16 and under (189). Thirdly, samples with missing information for any item were also deleted (10,572). Fourthly, to obtain the instrumental variable, samples from districts with a small population size were excluded (767). After this data-screening process, we finally obtained 17066 valid samples.

The key variables are defined in the following subsections.

### 2.1. Mental Health

This study used the simplified Center for Epidemiological Studies—Depression (CES-D) scale to measure mental health, which has shown good reliability and validity in the previous literature [45,46,47]. Respondents were asked about the frequency of the following behaviors or feelings in the past week: (1) “I didn’t feel depressed”, (2) “I found it easy to do anything”, (3) “I slept well”, (4) “I felt happy”, (5) “I didn’t feel lonely”, (6) “I live happily”, (7) “I didn’t feel sad”, (8) “I believe life can continue”. Each question is scored from 0 (5–7 days) to 3 (less than 1 day), and we added the scores to measure mental health. Thus, the variable of mental health ranged from 0 to 24, and the higher the value, the better the individual mental health.

### 2.2. Social Capital

This study measures individual social capital in terms of cognitive and structural social capital. Based on existing definitions and social capital questionnaires, we selected social help and social trust as the indicators of cognitive social capital. We measured social help by asking the question “Do you think most people are willing to help others?” [14], and measured social trust by asking “Do you think most people are trustworthy?” [48,49]; both questions took 1 if the respondents thought so, and 0 otherwise. We selected social networks and social participation as the indicators of structural social capital. We measured social networks by the logarithm of gift expenditure, since, in Chinese culture, social communication is often accompanied by mutual gifting; thus, gifting expenditure can reflect the depth and breadth of individual social networks [24,50,51]. We measured social participation by the question “How many memberships do you hold in the Communist Party of China, the Communist Youth League, the Trade Union, or the Workers’ Association?”, for which the responses ranged from 0 (none of them) to 4 (all of them) [14]. The higher the value, the greater the degree of social participation.

### 2.3. Control Variables

Other factors, such as individual characteristics and behavior, were included. Individual characteristics included age, gender, education, income, urbanicity, marital status, medical expenditure, and job type. Individual behaviors included smoking, reading, alcoholism, taking a noon break, and exercising. In addition, family characteristics included fuel and water for cooking, per capita income, and size.

Table 1 presents the summary statistics for all those variables.

## 3. Empirical Approach

Following the previous literature, mental health is a continuous variable, so we used the following OLS model:

(1) $$MH_{i}=\beta_{0}+\overset{4}{\underset{j=1}{\sum }}\beta_{j1}SC_{ji}+\overset{17}{\underset{k=1}{\sum }}\beta_{k2}{\mathrm{Control}}_{ki}+\varepsilon_{i}$$

where *MH_i_* is the mental health of individual *i*, $SC_{ji}$ (*j* = 1,2,3,4) is social trust ($SC_{1i}$*),* social help ($SC_{2i}$), social participation ($SC_{3i}$), and gifting expenditure ($SC_{4i}$*)* of individual *i*, respectively. ${\mathrm{Control}}_{ki}$ (*k* = 1,2,...,17) is gender, age, marital status, education, job type, income, medical expenditure, noon break, smoking, alcoholism, reading, exercise, family size, family income, cooking fuel, and water of individual *i*, respectively, $\beta_{j1},\beta_{k2}$ are the corresponding coefficients, and $\varepsilon_{i}$ is the error term.

In this model, in order to reduce the errors caused by endogeneity, we also consider the social and economic characteristics of individuals and families, including gender, age, education, income, medical expenditure, family size, family income, etc., because these variables have been addressed to have significant impacts on mental health in existing studies. In addition, some studies have also found a significant relationship between living behaviors and residents’ health, so we also take it as a part of the control variable.

For this model, there may be endogeneity, mainly arising from reverse causality. Social capital may affect mental health through individual feelings and available resources. On the other hand, individuals with higher mental health are likely to participate in social interactions and overestimate their social position, and thus obtain higher social capital [52]. To solve this problem, we used an instrumental variable (IV) and two-stage least square (TSLS) regression. In particular, this study considered the average social capital level of the 223 districts as the instrumental variable. We would like to discuss the following assumptions for valid IVs [53].

Exclusion Restriction Assumption: Individual mental health is not affected by the average level of social capital of the district once individual social capital is taken into account. On the one hand, the instrumental variables in this paper, i.e., the average level of social capital of a district, is different from the community social capital in the previous literature. The existing studies have addressed the association between community social capital and individual mental health, which may lead to the violation of exclusion restriction [20,54]. The previous literature mostly measures community social capital from community belongingness, infrastructure construction, participation, support, and community-based occupations, and analyzes its impact on residents’ mental health [55,56]; little literature addressed the significant impact of individual social capital at the cluster level on mental health. On the other hand, social capital measured at the cluster level has also been used as an instrumental variable in the existing literature; for example, the research on the impact of social capital on women empowerment [57], and the impact of social security polices [58], which also supports the validity of IV at the cluster lever. Moreover, since the number of IVs is equal to the number of endogenous variables, i.e., the model is exactly identical, the instrumental variables can be considered as exogenous from the perspective of statistical methods.

Relevance Assumption: The average social capital at the cluster level of a district has a strong correlation with individual social capital. This is fulfilled since the generation shows the direct relationship between individual social capital and the instrumental variables. In order to make the IV estimation more reliable, we carried out a weak instrumental variable test in the two-stage least square regression, and as the result shown in Section 4.1, the assumption is fulfilled.

No Instrument–Outcome Confounder Assumption: There are no other confounding factors between the average level of individual social capital and mental health. As the previous literature addressed, personal and family characteristics have impacts on individual mental health, such as age, physical health, family population, income, etc. Some of these characteristics also have impacts on personal social capital, which may result in the violation. Those variables are observed, and we put them into the regression model to separate the confounding effects.

Monotonicity Assumption: There is no one who would have lower social capital if living in a district with high average social capital, but have higher social capital if living in a district with low average social capital. On the one hand, the average social capital level is generated based on individual social capital. Therefore, individuals living in a district with a higher/lower average social capital level have a greater probability of higher/lower individual social capital. On the other hand, a district with higher average social capital is more likely to have better cultural, economic, and social foundation conditions to promote the accumulation of individual social capital, and vice versa. Therefore, the IV fulfills the assumption.

We used the following TSLS model to carry out an analysis that included the endogenous variables for social capital [59,60]:

(2) $$SC_{ji}=\alpha \prime_{1}+\overset{4}{\underset{l=1}{\sum }}\beta \prime_{l1}{\mathrm{IV}}_{li}+\overset{17}{\underset{k=1}{\sum }}\beta \prime_{k2}Controls_{ki}+\varepsilon_{i}$$

(3) $${MH}_{i}=\alpha +\overset{4}{\underset{j=1}{\sum }}\beta_{j1}SC_{ji}+\overset{17}{\underset{k=1}{\sum }}\beta_{k2}Controls_{ki}+\varepsilon_{i}$$

where ${\mathrm{IV}}_{li}$ (l = 1,2,3,4) is the average level of social trust (${\mathrm{IV}}_{1i}$), social help (${\mathrm{IV}}_{2i}$), social participation (${\mathrm{IV}}_{3i}$), and gifting expenditure (${\mathrm{IV}}_{4i}$) of the district. $SC_{ji}$(*j* = 1,2,3,4) and ${\mathrm{Control}}_{ki}$ (*k* = 1,2,...,17) have the same meanings as in Equation (1). Equation (2) estimates the relationships among the instrumental variables, the control variables, and social capital. Equation (3) estimates the impacts of social capital and other variables on mental health, considering the instrumental variables to obtain more accurate results.

In order to check the robustness of the results that we obtained through the two-step least squares (2SLS) model, we carried out two robustness checks. Firstly, we added two new variables, i.e., faith and entertainment expenditure, which may have impacts on mental health, to the basic model to observe the change in the relationship between social capital and mental health. Second, we analyzed the sample that only included individuals in the labor market to check the robustness of the impact of social capital on mental health in the alternative sample.

In addition, in order to study the heterogeneity of the impact of social capital on mental health, we also carried out heterogeneity analysis. Specifically, we conducted subgroup regression based on urbanicity, gender, age, and geographical area, and carried out permutation tests for the coefficients between groups [61].

## 4. Results

### 4.1. Regression

There was no problem of collinearity in the model (see the Appendix A for the results of the collinearity test).

Table 2 shows the OLS and IV-2SLS results for the effects of social capital on mental health. As indicated in Column 2, social trust (0.571), social help (1.002), social participation (−0.228), and gifting expenditure (0.120) have statistically significant impacts on mental health at the 1% level. Moreover, gender (0.919), age (0.007), marital status (1.028), education (0.202), medical expenditure (−0.195), smoking (−0.295), exercise (0.217), family size (0.081), per capita income (0.819), and cooking fuel (0.377) have statistically significant impacts on individual mental health at the 1% level, and individual income (0.04) and alcoholism (0.214) significantly affect mental health at the 5% level.

The IV-2SLS model in Column 3 differs from the OLS model. Social trust (1.854), social help (1.902), and gifting expenditure (0.185) have stronger impacts on mental health, while the impact of social participation on mental health is no longer significant. Additionally, gender (0.906), age (0.006), marital status (1.036), education (0.112), medical expenditure (−0.185), smoking (−0.244), alcoholism (0.165), exercise (0.16), family size (0.076), family per capita income (0.797), and cooking fuel (0.396) still have statistically significant impacts on individual mental health, but differed slightly from the results of the OLS model. In addition, individual income no longer had a significant impact on mental health in the TSLS model, and reading (−0.287) showed the opposite result.

According to the Durbin–Wu–Hausman test for endogeneity, the null hypothesis of the exogeneity of social trust, social help, gifting expenditure, and social participation is rejected at the 1% level in Column 2. Thus, it is reasonable to use the IV-2SLS model. Moreover, the value of the F-statistic of the weak IV test is 84.56, higher than the general standard of 10; thus, the instrumental variables are useful.

As shown in Table 2, individual mental health is affected by social capital, which is different from previous studies showing no or little impact of gifting expenditure and social participation on mental health.

### 4.2. Robustness Check

#### 4.2.1. Adding Variables

Due to the complexity of individual mental health, the previous conclusions may be affected by missing variables. Therefore, we carried out a robustness check by adding variables. Specifically, we included the respondents’ faith and family entertainment expenditure. The faith variable took 1 if the respondent believes in God, Allah, ancestral spirits, etc., and 0 otherwise. The entertainment expenditure variable was the logarithm of household spending on entertainment.

As shown in Column 2 of Table 3, social trust (1.856) and social help (1.900) still have a statistically significant impact on mental health at the 1% level, and gifting expenditure (0.185) significantly affected mental health at the 5% level. Additionally, gender (0.91), age (0.006), marital status (1.036), education (0.112), medical expenditure (−0.185), smoking (−0.244), alcoholism (0.165), reading (−0.290), exercise (0.160), family size (0.076), per capita income (0.796), and cooking fuel (0.397) had a significant impact on mental health. In addition, individual faith and family entertainment expenditure had no significant impact on mental health.

By adding variables into the basic model, we showed that the results for the impacts of social trust, social help, and gifting expenditure on mental health are robust.

#### 4.2.2. Alternate Sample

Individuals who are employed are more likely to accumulate social capital at a faster rate. Therefore, we excluded the respondents who had withdrawn from the labor market at the time of the survey to carry out a robustness check.

As shown in Column 3 of Table 3, 15,136 working samples were used for the regression. Social trust (2.124), social help (2.042), and gifting expenditure (0.225) still had a significant positive impact on mental health. In addition, gender (0.912), age (0.008), marital status (0.96), education (0.11), medical expenditure (−0.178), smoking (−0.230), alcoholism (0.156), reading (−0.334), family size (0.075), family per capita income (0.779), and cooking fuel (0.385) had a significant impact on mental health.

According to the alternate sample, the results showing the positive impact of social capital on mental health are robust.

### 4.3. Heterogeneity

#### 4.3.1. Urbanicity

In China, urban areas are ahead of rural areas in terms of economy, culture, infrastructure construction, etc. Although the previous analysis did not find that urbanicity has an significant impact on individual mental health, there may be heterogeneity for the impact of social capital on mental health. Using China’s urbanicity system and the survey information, we divided the samples into the urban group and the rural group, with sample sizes of 3638 and 13428, respectively.

As shown in Table 4, social trust (2.244) and gifting expenditure (0.241) had a positive and significant impact on the mental health of rural residents, but had no significant impact on that of urban residents. Social participation (1.637) significantly improved the mental health of urban residents, but had no significant impact on that of rural residents. Social trust significantly improved the mental health of rural (1.879) and urban (2.493) residents. The permutation test shows that at the significance of the 1% level, the impact of social trust on the mental health of rural residents is significantly higher than that of urban residents, while the impact of social participation on the mental health of urban residents is significantly higher than that of rural residents.

In addition, education (0.196), income (0.038), smoking (−0.288), alcoholism (0.215), reading (−0.243), exercise (0.172), and cooking fuel (0.392) had a significant impact on the mental health of rural residents, but no significant impact on that of urban residents, while age (0.017) and cooking water (0.578) only significantly affected the mental health of urban residents. Additionally, marital status, medical expenditure and family income had a significant impact on the mental health of urban residents (0.863, −0.173, and 0.686, respectively) and rural residents (1.06, −0.188, and 0.759, respectively). Gender and family size significantly affected the mental health of both urban residents (0.92 and 0.09, respectively) and rural residents (0.891 and 0.072, respectively). As shown in the permutation test, age, job type, and cooking water have significantly stronger impacts on the mental health of the urban residents, while education, income, and taking a noon break have contrary impacts.

#### 4.3.2. Gender

There has been a patriarchal ideology in Chinese culture for a long time, which has resulted in females’ weak position in daily life. There may be heterogeneity in gender for the impact of social capital on mental health. This study thus divided the sample into females and males, with a sample size of 8357 and 8691, respectively.

As shown in Table 5, social trust and social help had a significant positive impact on the mental health of females (1.818 and 1.896, respectively) and males (1.924 and 1.886, respectively). Gifting expenditure (0.302) significantly improved females’ mental health but had no significant impact on that of males. Social participation had no significant impact on the mental health of males or females. As shown in column 4, at the significance of the 10% level, the impact of social participation on men’s mental health is significantly stronger than that of women, while the impacts of other dimensions of social capital on mental health has no obvious gender heterogeneity.

Moreover, education (0.177) and exercise (0.186) significantly affected females’ mental health, but had no significant impact on that of males. Age (0.01), smoking (−0.281), and alcoholism (0.156) only had a significant impact on males’ mental health. Marital status, medical expenditure, reading, family size, per capita income, and cooking fuel had a significant impact on the mental health of males (1.243, −0.165, −0.229, 0.064, 0.75, and 0.235, respectively) and females (0.669, −0.207, −0.393, 0.088, 0.889, and 0.568, respectively). In addition, age and marital status have significantly stronger impacts on men’s mental health than they do on women, while education, medical expenditure and cooking fuel have the opposite impacts.

#### 4.3.3. Age

Individuals at different life stages have different conditions, both physically and psychologically. Thus, there may be heterogeneity in terms of age for the impact of social capital on mental health. According to the standards of the United Nations, we defined those aged 17–44 years as the young group, those aged 45–59 years as the middle-aged group, and those over 60 years old as the older group, with sample sizes of 6957, 6278, and 3831, respectively.

As shown in Table 6, social trust has a significant impact on the mental health of young people (1.816), middle-aged people (2.025), and older people (1.587). Social help has a significant impact on the mental health of young people (2.305) and middle-aged people (1.736), but has no significant impact on that of older people. Social participation only significantly improved the mental health of middle-aged people (0.357), but had no significant impact on that of other groups. Moreover, we find that the impact of social help on the mental health of young people is significantly stronger than that of middle-aged people, while social participation is on the contrary. The impact of gifting expenditure on the mental health of middle-aged people is significantly stronger than that of young people; meanwhile, that of the young people is significantly stronger than that of the older people.

In addition, individual income (0.064) only has an significant impact on the mental health of the middle-aged group, while education (0.202) and alcoholism (0.383) only significantly affect the mental health of older people. Smoking and reading significantly affect the mental health of young people (−0.355 and −0.258, respectively) and middle-aged people (−0.280 and −0.547, respectively). Gender (0.827, 1.013, and 0.897, respectively), marital status (0.924, 1.334, and 0.931, respectively), medical expenditure (−0.179, −0.21, and −0.139, respectively), noon break (−0.179, −0.210, and −0.139, respectively), family size (0.055, 0.082, and 0.085, respectively), family income (0.836, 0.781, and 0.73, respectively), and cooking fuel (0.41, 0.416, and 0.365, respectively) have significant impacts on mental health in all age groups. The impacts of marital status on the mental health of middle-aged people is significantly stronger than that of the older people, and that of the latter is significantly stronger than that of young people. The effect of education on the mental health of middle-aged people is significantly weaker than that of young and older people, and the impact of job type on mental health of the older people is significantly stronger than that of the young and the middle-aged people. Additionally, the impacts of family income and cooking fuel on the mental health of young people are significantly higher than that of middle-aged people.

#### 4.3.4. Geographic Location

China’s development has produced gaps between geographic locations. Briefly, the provinces along the eastern coast precede other provinces. Therefore, to analyze the heterogeneity by geographic location of the impact of social capital on mental health, we defined the samples from Beijing, Tianjin, Hebei, Shandong, Zhejiang, Shanghai, Jiangsu, Fujian, Guangdong, and Hainan as the eastern group; the samples from Shanxi, Henan, Hubei, Hunan, Jiangxi, and Anhui as the middle group; the samples from Heilongjiang, Liaoning, and Jilin as the northeast group; and the samples from Chongqing, Sichuan, Guangxi, Guizhou, Yunnan, Shaanxi, Gansu, Inner Mongolia, Ningxia, Xinjiang, Qinghai, and Tibet as the western group, with sample sizes of 5401, 4094, 2284, and 5287, respectively.

As shown in Table 7, social trust had a significant positive impact on the mental health of the eastern group (2.564) and the middle group (3.149). Social help has a significant impact on the middle group (2.811), the northeast group (2.210), and the western group (2.146). In addition, the impact of social trust on the mental health of residents in the east is significantly stronger than that of the northeast residents, while that of residents in the west is significantly lower than that in other geographic locations. The impact of social help on the mental health of residents in the northeast is significantly stronger than that of residents in the west. The impact of gifting expenditure on the mental health of residents in the east is significantly stronger than that of the middle, and that of the residents in the west is significantly stronger than that in the northeast. The promotion effect of social participation on the mental health of residents in the northeast is significantly stronger than that in the east and west. Meanwhile, that of the residents in the middle is stronger than that in the west.

In addition, gender and marital status had significant impacts on the mental health of all groups, while other variables had different significant impacts on some groups (see Table 7 for details). The impacts of age, marital status, urbanicity, education, job type, medical expenditure, smoking, reading, family income, cooking fuel, and water on mental health shows heterogeneity in geographic location (see Table 8 for details).

## 5. Discussion

To our knowledge, this study has expanded the research on the impact of social capital on mental health in a large representative sample covering the whole of China, in particular the causal inference for the impact of social capital on mental health. Our results support the important impact of social capital on mental health, which will help to formulate social policies to promote residents’ mental health.

To solve the problem of endogeneity in the model, we established the average level of social capital of a district as the instrumental variable and applied the two-stage least squares (2SLS) method. We found that social capital significantly improved residents’ mental health. Specifically, cognitive social capital, measured as social trust and social help, had a significant positive impact on individuals’ mental health. However, the impact of structural social capital on mental health varied across different dimensions. Social networks, measured by gifting expenditure, had a significant impact on individuals’ mental health, but social participation had no significant impact. We checked that these results were robust by using additional variables and alternative samples. These findings have policy and intervention implications. Social capital can be used as one of the tools to improve residents’ mental health. Specifically, community health policies should pay more attention to improving cognitive social capital, and encourage residents to establish mutual aid organizations, such as women’s federations, elderly associations, volunteer associations, etc. In addition, it is necessary to enhance the interaction between community residents, families, residents and village (neighborhood) committee cadres, for example, family fellowship activities, and meetings between civil servants and residents, to improve residents’ sense of trust and mutual help. For structural social capital, community policies should be biased towards family cultural guidance and community infrastructure construction to encourage individuals to expand social networks reasonably.

Another main contribution of this study is that we found heterogeneity in the impact of social capital on individual mental health by urbanicity, gender, age, and geographic location, which indicates that different health promotion policies should be implemented for different groups. For almost all groups, mental health is significantly and positively affected by social capital, which indicates that social capital can be used as an effective tool to improve the mental health of different groups based on subdimensions of social capital and group characteristics.

In addition, we also found that gender, age, education, marital status, alcoholism, exercise, family size, family per capita income, and cooking fuel had significantly positive effects on individuals’ mental health, while medical expenditure, smoking, and reading had opposite significant impacts. These findings suggest that individual and family-based mental health promotion policies are still necessary. Mental health monitoring and guarantee policies for women, young people, unmarried people, people with a low education level, and people with physical limitations need to be strengthened. Meanwhile, through the promotion of culture and a community environment, policies can guide residents to form good living habits, gradually transferring the functions of mutual assistance and belonging from the family to the community.

There are some open problems following this study. First, following Arezzo et al. [62], Fiorillo [25], Phyllis [57], Fang [38], Sun and Lu [14], and Kilian et al. [30], we used cross-sectional data for causal inference. See, for example, Reichenheim et al. [63], for the conditions for causal inference with cross-sectional data. Indeed, at least one theoretical analysis for causal inference with cross-sectional data is important in future research. Second, this research mainly studied the existence of the impact of social capital on mental health, and introducing intermediary variables and regulatory variables to analyze the paths will be an important direction in future research.

## 6. Conclusions

Based on the CFPS 2018 data, this study applied the IV-2SLS method to analyze the impact of social capital on mental health and carried out robustness checks and heterogeneity analyses. We found that social trust, social help, and social networks (gifting expenditure) had significant positive impacts on individuals’ mental health, and these impacts are different due to the differences in the subdimensions of social capital and group characteristics. The findings indicate that social capital should be given more attention when formulating mental health-promotion policies. Future work should be carried out to clarify the impact mechanism and the path of social capital on mental health.

## Figures and Tables

**Table 1:** Summary statistics.

Variable and Value	Definition	N/Mean	%/Std
Mental Health	Summation of CES-D	18.341	0.993
Social Trust	Do you think most people are trustworthy?		
0	No	7707	45.2
1	Yes	9359	54.8
Social Help	Do you think most people are willing to help others?		
0	No	5337	31.3
1	Yes	11,729	68.7
Social Participation	How many memberships do you hold in the Communist Party of China, the Communist Youth League, the Trade Union, or the Workers’ Association?		
0	None of them	12,854	75.3
1	One of them	3716	21.8
2	Two of them	439	2.6
3	Three of them	51	0.3
4	All of them	6	0.03
Gifting Expenditure	Logarithm of gifting expenditure	3.210	0.993
Gender	Gender of respondents	0.511	0.500
0	Female	8375	49.1
1	Male	8691	50.9
Age	Age of the respondents	47.353	14.218
Marital status	What is your marital status?		
0	In a relationship	2424	14.2
1	Not in a relationship	14,642	85.8
Urbanicity	What is your urbanicity?		
0	Rural	13,428	78.7
1	Urban	3638	21.3
Education	What is your education level?		
1	Illiterate	4000	23.4
2	Primary school	3824	22.4
3	Junior middle school	5082	29.8
4	High school	2282	29.8
5	Junior college	1833	10.7
6	College and above	45	0.3
Job type	What is the nature of your job?		
0	Agricultural	8105	47.5
1	Non-agricultural	8961	52.5
Income	Logarithm of individual income	2.601	2.052
Medical Expenditure	Logarithm of medical expenditure	4.790	3.463
Noon Break	Do you take a noon break?		
0	No	7936	46.5
1	Yes	9130	53.5
Smoking	Do you smoke?		
0	No	9618	56.4
1	Yes	7448	43.6
Alcoholism	Have you been drunk more than three times in the past week?		
0	No	14,217	83.3
1	Yes	2849	16.7
Reading	Do you read books, magazines, newspapers, and so on?		
0	No	13,351	78.2
1	Yes	3715	21.8
Exercise	Do you exercise for more than an hour a day?		
0	No	8287	54.4
1	Yes	7779	45.6
Family Size	Number of people in the family	4.291	2.042
Family Income	Logarithm of per capita income of the household	4.150	0.452
Cooking Fuel	What kind of fuel does your family use for cooking?		
0	Firewood or coal	5281	30.9
1	Gas, natural gas, solar, biogas, or electricity	11,785	69.1
Cooking Water	What kind of water does your family use for cooking?		
0	River and lake water, well water, rainwater, cistern water, or pond water	4732	27.7
1	Tap water or bottled water	12,334	72.3

Note: Based on CFPS 2018. The table reports the frequency and percentage for binary variables, and the mean and standard deviation for continuous variables.

**Table 2:** Regression results.

Variables	(1)	(2)
	OLS	TSLS
Social Trust	0.571 ***	1.854 ***
	(0.063)	(0.398)
Social Help	1.002 ***	1.902 ***
	(0.067)	(0.423)
Gifting Expenditure	0.120 ***	0.185 **
	(0.030)	(0.085)
Social Participation	−0.126 ***	0.111
	(0.059)	(0.422)
Gender	0.919 ***	0.906 ***
	(0.092)	(0.097)
Age	0.007 ***	0.006 **
	(0.003)	(0.003)
Marital Status	1.028 ***	1.036 ***
	(0.087)	(0.102)
Urbanicity	0.038	0.042
	(0.083)	(0.102)
Education	0.202 ***	0.112 **
	(0.032)	(0.051)
Job Type	0.046	0.094
	(0.085)	(0.088)
Income	0.040 **	0.027
	(0.019)	(0.020)
Medical Expenditure	−0.195 ***	−0.185 ***
	(0.009)	(0.009)
Noon Break	0.063	0.041
	(0.059)	(0.061)
Smoking	−0.295 ***	−0.244 **
	(0.091)	(0.095)
Alcoholism	0.214 **	0.165 *
	(0.083)	(0.087)
Reading	−0.094	−0.287 ***
	(0.080)	(0.100)
Exercise	0.217 ***	0.160 **
	(0.060)	(0.069)
Family Size	0.081 ***	0.076 ***
	(0.015)	(0.016)
Family Income	0.819 ***	0.797 ***
	(0.078)	(0.090)
Cooking Fuel	0.377 ***	0.396 ***
	(0.070)	(0.073)
Cooking Water	0.091	0.053
	(0.068)	(0.071)
Constant	11.460 ***	10.310 ***
	(0.365)	(0.459)
Endogeneity Test (*p*-value)		0.000
Weak IV Test (F-statistics)		84.560
N	17,066	17,066
R^2^	0.118	0.069

Note: Based on CFPS 2018.The betas are reported on the same line as the variable name, and robust standard errors are shown in parentheses. *** *p* < 0.01, ** *p* < 0.05, * *p* < 0.1.

**Table 3:** Robustness check.

Variables	(1)	(2)
Social Trust	1.856 ***	2.124 ***
	(0.398)	(0.446)
Social Help	1.900 ***	2.042 ***
	(0.423)	(0.470)
Gifting Expenditure	0.185 **	0.225 *
	(0.085)	(0.090)
Social Participation	0.120	−0.011
	(0.423)	(0.432)
Gender	0.910 ***	0.912 ***
	(0.097)	(0.102)
Age	0.006 **	0.008 *
	(0.003)	(0.003)
Marital Status	1.036 ***	0.960 ***
	(0.102)	(0.108)
Urbanicity	0.040	−0.013
	(0.102)	(0.112)
Education	0.112 **	0.110 *
	(0.051)	(0.054)
Job Type	0.094	0.063
	(0.088)	(0.097)
Income	0.027	0.032
	(0.020)	(0.022)
Medical Expenditure	−0.185 ***	−0.178 ***
	(0.009)	(0.010)
Noon Break	0.041	0.020
	(0.061)	(0.065)
Smoking	−0.244 **	−0.230 *
	(0.095)	(0.100)
Alcoholism	0.165 *	0.156 *
	(0.087)	(0.091)
Reading	−0.290 ***	−0.334 ***
	(0.100)	(0.106)
Exercise	0.160 **	0.149 *
	(0.069)	(0.073)
Family Size	0.076 ***	0.075 ***
	(0.016)	(0.017)
Family Income	0.796^***^	0.779 ***
	(0.090)	(0.098)
Cooking Fuel	0.397 ***	0.385 ***
	(0.073)	(0.077)
Cooking Water	0.052	0.026
	(0.071)	(0.075)
Faith	−0.027	
	(0.070)	
Entertainment Expenditure	0.011	
	(0.010)	
Constant	10.320 ***	10.090 ***
	(0.461)	(0.510)
N	17,066	15,136
R^2^	0.069	0.042

Note: Based on CFPS 2018. The betas are reported on the same line as the variable name, and robust standard errors are shown in parentheses. *** *p* < 0.01, ** *p* < 0.05, * *p* < 0.1.

**Table 4:** Heterogeneity by urbanicity.

Variables	Rural	Urban	Permutation Test
Social Trust	2.244 ***	−0.358	0.008 ***
	(0.442)	(0.982)	
Social Help	1.879 ***	2.493 **	0.297
	(0.472)	(1.008)	
Gifting Expenditure	0.241 **	−0.007	0.113
	(0.097)	(0.183)	
Social Participation	−0.665	1.637 **	0.010 ***
	(0.526)	(0.731)	
Gender	0.891 ***	0.920 ***	0.444
	(0.115)	(0.187)	
Age	0.003	0.017 ***	0.025 **
	(0.003)	(0.007)	
Marital Status	1.060 ***	0.863 ***	0.218
	(0.122)	(0.202)	
Education	0.196 ***	−0.091	0.011 **
	(0.056)	(0.124)	
Job Type	0.023	0.365	0.063 *
	(0.099)	(0.235)	
Income	0.038 *	−0.031	0.081 *
	(0.022)	(0.056)	
Medical Expenditure	−0.188 ***	−0.173 ***	0.236
	(0.011)	(0.018)	
Noon Break	0.105	−0.201	0.026 **
	(0.071)	(0.129)	
Smoking	−0.288 **	−0.276	0.471
	(0.112)	(0.188)	
Alcoholism	0.215 **	−0.018	0.152
	(0.102)	(0.175)	
Reading	−0.243 **	−0.171	0.377
	(0.120)	(0.179)	
Exercise	0.172 **	0.176	0.467
	(0.080)	(0.147)	
Family Size	0.072 ***	0.090 **	0.320
	(0.018)	(0.039)	
Family Income	0.759 ***	0.686 ***	0.401
	(0.102)	(0.208)	
Cooking Fuel	0.392 ***	0.281	0.254
	(0.078)	(0.254)	
Cooking Water	0.020	0.578 **	0.002 ***
	(0.076)	(0.259)	
Constant	10.220 ***	11.620 ***	0.140
	(0.521)	(1.136)	
N	13,428	3638	
R^2^	0.041	0.016	

Note: Based on CFPS 2018. The betas are reported on the same line as the variable name, and robust standard errors are shown in parentheses. *** *p* < 0.01, ** *p* < 0.05, * *p* < 0.1. Column 4 reports the *p*-value of the Permutation test.

**Table 5:** Heterogeneity by gender.

Variables	Female	Male	Permutation Test
Social Trust	1.818 ***	1.924 ***	0.451
	(0.561)	(0.561)	
Social Help	1.896 ***	1.886 ***	0.461
	(0.609)	(0.591)	
Gifting Expenditure	−0.380	0.514	0.147
	(0.653)	(0.558)	
Social Participation	0.302 **	0.065	0.079 *
	(0.124)	(0.116)	
Age	−0.002	0.010 ***	0.026 **
	(0.004)	(0.004)	
Marital Status	0.669 ***	1.243 ***	0.004 ***
	(0.169)	(0.132)	
Urbanicity	−0.078	0.127	0.150
	(0.138)	(0.149)	
Education	0.177 **	0.008	0.045 **
	(0.074)	(0.070)	
Job Type	0.071	0.092	0.451
	(0.135)	(0.116)	
Income	0.029	0.027	0.499
	(0.032)	(0.027)	
Medical Expenditure	−0.207 ***	−0.165 ***	0.007 ***
	(0.014)	(0.012)	
Noon Break	0.105	−0.047	0.108
	(0.089)	(0.085)	
Smoking	−0.214	−0.281 ***	0.322
	(0.199)	(0.108)	
Alcoholism	0.283	0.156*	0.227
	(0.243)	(0.093)	
Reading	−0.393 **	−0.229 *	0.175
	(0.157)	(0.129)	
Exercise	0.186 *	0.145	0.401
	(0.098)	(0.098)	
Family Size	0.088 ***	0.064 ***	0.223
	(0.023)	(0.022)	
Family Income	0.889 ***	0.750 ***	0.255
	(0.132)	(0.124)	
Cooking Fuel	0.568 ***	0.235 **	0.017 **
	(0.106)	(0.099)	
Cooking Water	0.039	0.103	0.392
	(0.103)	(0.097)	
Constant	10.170 ***	11.690 ***	0.066*
	(0.702)	(0.605)	
N	8375	8691	
R^2^	0.070	0.037	

Note: Based on CFPS 2018. The betas are reported on the same line as the variable name, and robust standard errors are shown in parentheses. Column 4 reports the *p*-value of the Permutation test. *** *p* < 0.01, ** *p* < 0.05, * *p* < 0.1.

**Table 6:** Heterogeneity by age.

Variables	Young	Middle-Aged	Older	Young and Older	Young and Middle-Aged	Middle-Aged and Older
Social Trust	1.816 ***	2.025 ***	1.587 **	0.137	0.343	0.192
	(0.644)	(0.667)	(0.805)			
Social Help	2.305 ***	1.736 **	1.321	0.364	0.078 *	0.191
	(0.647)	(0.720)	(0.923)			
Gifting Expenditure	0.234	0.591	−0.607	0.053 *	0.063 *	0.386
	(0.700)	(0.664)	(0.850)			
Social Participation	0.069	0.357 ***	0.044	0.119	0.024 *	0.376
	(0.143)	(0.133)	(0.173)			
Gender	0.827 ***	1.013 ***	0.897 ***	0.234	0.330	0.336
	(0.150)	(0.160)	(0.207)			
Urbanicity	0.105	0.017	0.113	0.260	0.134	0.370
	(0.163)	(0.165)	(0.215)			
Marital Status	0.924 ***	1.334 ***	0.931 ***	0.028 **	0.403	0.046 **
	(0.162)	(0.160)	(0.205)			
Education	0.088	0.001	0.202 **	0.460	0.038 **	0.056 *
	(0.076)	(0.083)	(0.096)			
Job Type	−0.090	0.079	0.227	0.095 *	0.411	0.063 *
	(0.134)	(0.142)	(0.176)			
Income	0.011	0.064 *	0.005	0.374	0.482	0.400
	(0.032)	(0.034)	(0.042)			
Medical Expenditure	−0.179 ***	−0.210 ***	−0.139 ***	0.458	0.373	0.394
	(0.015)	(0.015)	(0.019)			
Noon Break	−0.179 ***	−0.210 ***	−0.139 ***	0.362	0.455	0.306
	(0.015)	(0.015)	(0.019)			
Smoking	−0.355 **	−0.280 *	0.0247	0.216	0.404	0.269
	(0.146)	(0.157)	(0.207)			
Alcoholism	0.189	0.042	0.383 **	0.406	0.142	0.115
	(0.136)	(0.146)	(0.188)			
Reading	−0.258 *	−0.547 ***	−0.007	0.153	0.133	0.494
	(0.157)	(0.164)	(0.202)			
Exercise	0.175	0.179	0.183	0.117	0.163	0.365
	(0.107)	(0.113)	(0.146)			
Family Size	0.055 **	0.082 ***	0.085 ***	0.087 *	0.426	0.128
	(0.025)	(0.027)	(0.032)			
Family Income	0.836 ***	0.781 ***	0.730 ***	0.275	0.076 *	0.251
	(0.146)	(0.144)	(0.192)			
Cooking Fuel	0.410 ***	0.416 ***	0.365 **	0.457	0.207	0.203
	(0.113)	(0.122)	(0.151)			
Cooking Water	0.043	−0.093	0.303 **	0.152	0.003 ***	0.141
	(0.112)	(0.118)	(0.147)			
Constant	10.920 ***	10.150 ***	11.300 ***	0.258	0.200	0.470
	(0.661)	(0.688)	(0.921)			
N	6957	6278	3831			
R^2^	0.052	0.073	0.090			

Note: Based on CFPS 2018. The betas are reported on the same line as the variable name, and robust standard errors are shown in parentheses. Columns 4–6 reports the *p*-value of the permutation test. *** *p* < 0.01, ** *p* < 0.05, * *p* < 0.1.

**Table 7:** Heterogeneity by geographic location.

Variables	Eastern	Middle	Northeast	West
Social Trust	2.564 ***	3.149 ***	0.784	0.831
	(0.851)	(0.919)	(0.990)	(0.623)
Social Help	0.942	2.811 ***	2.210 *	2.146 ***
	(0.798)	(0.926)	(1.279)	(0.711)
Gifting Expenditure	0.394	−0.568	0.005	0.325
	(0.774)	(1.004)	(1.133)	(0.710)
Social Participation	0.142	0.189	0.257	0.177
	(0.152)	(0.192)	(0.228)	(0.150)
Gender	0.928 ***	0.777 ***	0.828 ***	1.073 ***
	(0.174)	(0.214)	(0.248)	(0.178)
Age	0.002	0.013 **	−0.003	0.010 *
	(0.005)	(0.006)	(0.008)	(0.005)
Marital Status	1.089 ***	1.104 ***	1.251 ***	0.874 ***
	(0.184)	(0.234)	(0.285)	(0.174)
Urbanicity	−0.084	0.205	−0.295	0.193
	(0.165)	(0.246)	(0.292)	(0.185)
Education	0.084	0.081	0.207	0.113
	(0.095)	(0.125)	(0.131)	(0.084)
Job Type	0.285*	0.431 **	−0.268	−0.177
	(0.161)	(0.194)	(0.233)	(0.160)
Income	0.002	−0.037	0.055	0.072 **
	(0.038)	(0.047)	(0.055)	(0.036)
Medical Expenditure	−0.168 ***	−0.175 ***	−0.208 ***	−0.198 ***
	(0.016)	(0.021)	(0.026)	(0.016)
Noon Break	0.082	−0.027	0.060	0.044
	(0.109)	(0.136)	(0.165)	(0.110)
Smoking	−0.568 ***	−0.159	−0.053	−0.129
	(0.170)	(0.207)	(0.245)	(0.176)
Alcoholism	0.099	0.288	0.162	0.126
	(0.153)	(0.194)	(0.242)	(0.156)
Reading	−0.492 ***	−0.203	−0.393	−0.076
	(0.171)	(0.215)	(0.331)	(0.176)
Exercise	0.102	0.195	0.385 **	0.097
	(0.124)	(0.153)	(0.169)	(0.127)
Family Size	0.051 *	0.103 ***	0.054	0.091 ***
	(0.028)	(0.035)	(0.045)	(0.029)
Family Income	0.586 ***	1.202 ***	0.884 ***	0.685 ***
	(0.167)	(0.210)	(0.244)	(0.155)
Cooking Fuel	0.442 ***	0.322 **	0.270	0.502 ***
	(0.128)	(0.160)	(0.196)	(0.132)
Cooking Water	0.018	0.179	−0.017	−0.001
	(0.125)	(0.162)	(0.191)	(0.127)
Constant	12.04 ***	7.012 ***	10.37 ***	10.85 ***
	(0.862)	(1.069)	(1.156)	(0.789)
N	5401	4094	2284	5287
R^2^	0.043		0.107	0.090

Note: Based on CFPS 2018. The betas are reported on the same line as the variable name, and robust standard errors are shown in parentheses. *** *p* < 0.01, ** *p* < 0.05, * *p* < 0.1. The R^2^ of the 2SLS results for the middle-area samples is negative, so they have been omitted, and we obtained more accurate regression coefficients.

**Table 8:** Permutation test.

Variables	East–Northeast	East–Middle	East–West	Middle–Northeast	Middle–West	Northeast–West
Social Trust	0.077 *	0.335	0.043 **	0.032 **	0.442	0.029 **
Social Help	0.391	0.115	0.139	0.219	0.119	0.023 **
Gifting Expenditure	0.344	0.012 **	0.500	0.082 *	0.347	0.048 **
Social Participation	0.020 **	0.169	0.450	0.273	0.005 ***	0.008 ***
Gender	0.495	0.113	0.302	0.169	0.173	0.435
Age	0.061 *	0.467	0.153	0.105	0.000 ***	0.069 *
Marital Status	0.466	0.405	0.207	0.408	0.026 **	0.039 **
Urbanicity	0.324	0.317	0.136	0.199	0.065 *	0.125
Education	0.182	0.021 **	0.430	0.199	0.276	0.308
Job Type	0.068 *	0.106	0.022 **	0.323	0.182	0.103
Income	0.431	0.148	0.079 *	0.199	0.285	0.336
Medical Expenditure	0.002 ***	0.413	0.109	0.004 ***	0.098 *	0.074 *
Noon Break	0.496	0.410	0.414	0.423	0.239	0.183
Smoking	0.408	0.474	0.026 **	0.360	0.453	0.322
Alcoholism	0.339	0.224	0.462	0.154	0.447	0.118
Reading	0.035 **	0.367	0.036 **	0.038 **	0.043 **	0.471
Exercise	0.309	0.230	0.489	0.442	0.275	0.326
Family Size	0.469	0.347	0.143	0.336	0.364	0.463
Family Income	0.241	0.402	0.337	0.355	0.031 **	0.026 **
Cooking Fuel	0.172	0.086 *	0.381	0.432	0.031 **	0.057 *
Cooking Water	0.059 *	0.053 *	0.473	0.347	0.038 **	0.096 *
Constant	0.029 **	0.135	0.178	0.190	0.122	0.409

Note: Based on CFPS 2018. The numbers are the *p*-value of the permutation test. *** *p* < 0.01, ** *p* < 0.05, * *p* < 0.1.

## Data Availability

Data used in this paper can be found from the China Family Panel Survey, http://www.isss.pku.edu.cn/cfps/ (accessed on 24 December 2021).

## Appendix A

The collinearity between variables may increase the regression error. This research carried out a collinearity test before the regression analysis. Tolerance and VIF values are the usual measures of collinearity. In general, the value of tolerance is between 0 and 1. The smaller the tolerance, the larger the VIF value and the more obvious the collinearity. When the VIF value is less than 10, there is no obvious collinearity in the regression model.

As shown in Table A1, gender had the highest VIF value (2.58) and the lowest tolerance (0.387), and noon break had the lowest VIF value (1.04) and the highest tolerance (0.961). All the VIF values of all variables in this model were less than 10 and the tolerances were close to 1. There is, therefore, no collinearity in this model.

**Table A1:** Collinearity.

Variable	VIF	Tolerance
Social Trust	1.190	0.841
Social Help	1.170	0.852
Gifting Expenditure	1.150	0.873
Social Participation	1.080	0.927
Gender	2.580	0.387
Age	1.770	0.565
Marital Status	1.130	0.888
Urbanicity	1.420	0.705
Education	2.090	0.479
Job Type	2.180	0.460
Income	1.780	0.561
Medical Expenditure	1.070	0.935
Noon Break	1.040	0.961
Smoking	2.450	0.408
Alcoholism	1.170	0.854
Reading	1.310	0.762
Exercise	1.090	0.921
Family Size	1.150	0.867
Family Income	1.520	0.658
Cooking Fuel	1.280	0.782
Cooking Water	1.130	0.888
Mean	1.460	0.750
N	17,066	

Note: Based on CFPS 2018.

## References

[1] Das-Munshi J., Lund C., Mathews C., Clark C., Rothon C., Stansfeld S. (2016). Mental Health Inequalities in Adolescents Growing Up in Post-Apartheid South Africa: Cross-Sectional Survey, SHaW Study. PLoS ONE.

[2] Wu H., Yin X. (2021). Review on the relationship between adolescent physique and mental health. Chin. J. Sch. Health.

[3] Wang X., Yang Y., Kong L., Tong T., Chen M. (2020). A Study on the Development Strategy for Children and Adolescents’ Sports Health Promotion in China. J. Chengdu Sport Univ..

[4] Yin X., Zeng Z. (2021). Bringing together physical and mental health and promoting positive youth development in China. Chin. J. Sch. Health.

[5] Gao L., Li S., Wu Z. (2019). Urban-Rural Differences in the Effect of Community Poverty on Elderly Mental Health—A Study Based on the 2014 Chinese Longitudinal Aging Social Survey. Popul. Dev..

[6] Xu F., Long H. (2020). Research on the relationship between poverty and mental health. Chin. J. Clin. Psychol..

[7] Shang Y., Shi Z. (2020). Rural-urban Migration and Migrants’ Mental Health: Based on the Analysis of CLDS Data. Northwest Popul. J..

[8] Liu G., Zhang W. (2020). Putonghua Proficiency and the Mental Health of Migrant Workers: An Empirical Study Based on Chinese General Social Survey. Appl. Linguist..

[9] Zhang C., Lan X. (2021). Community Support and Elderly Health Promotion. World Surv. Res..

[10] Vos T., Barber R.M., Bell B., Bertozzi-Villa A., Biryukov S., Bolliger I., Charlson F., Davis A., Degenhardt L., Dicker D. (2015). Global, regional, and national incidence, prevalence, and years lived with disability for 301 acute and chronic diseases and injuries in 188 countries, 1990–2013: A systematic analysis for the Global Burden of Disease Study 2013. Lancet.

[11] Huang Y., Wang Y., Wang H., Liu Z., Yu X., Yan J., Yu Y., Kou C., Xu X., Lu J. (2019). Prevalence of mental disorders in China: A cross-sectional epidemiological study. Lancet Psychiatry.

[12] Donkin A., Goldblatt P., Allen J., Nathanson V., Marmot M. (2017). Global action on the social determinants of health. Br. Med. J. Glob. Health.

[13] Bourdieu P. (1986). The Forms of Capital.

[14] Sun Q., Lu N. (2020). Social Capital and Mental Health among Older Adults Living in Urban China in the Context of COVID-19 Pandemic. Int. J. Environ. Res. Public Health.

[15] Han J., Jia P., Huang Y., Gao B., Yu B., Yang S., Yu J., Xiong J., Liu C., Xie T. (2020). Association between social capital and mental health among older people living with HIV: The Sichuan Older HIV-Infected Cohort Study (SOHICS). BMC Public Health.

[16] Lu N., Zhang J. (2019). Social Capital and Self-Rated Health among Older Adults Living in Urban China: A Mediation Model. Sustainability.

[17] Putnam R.D., Leonardi D.R. (1994). Making Democracy Work: Civic Traditions in Modern Italy. Contemp. Sociol..

[18] Gu N. (2020). The effects of neighborhood social ties and networks on mental health and well-being: A qualitative case study of women residents in a middle-class Korean urban neighborhood. Soc. Sci. Med..

[19] Li C., Shan J., Fang X. (2020). Effects of multi-dimensional social capital on mental health of children in poverty: An empirical study in Mainland China. J. Health Psychol..

[20] Morozumi R., Matsumura K., Hamazaki K., Tsuchida A., Takamori A., Inadera H., Kamijima M., Yamazaki S., Ohya Y., Kishi R. (2020). Impact of individual and neighborhood social capital on the physical and mental health of pregnant women: The Japan Environment and Children’s Study (JECS). BMC Pregnancy Childbirth.

[21] Miu X., Bian Y. (2020). Epidemic-Specific Social Capital, Physical Activity and Health Status. J. Shanghai Univ. Sport.

[22] Nyqvist F., Forsman A.K., Giuntoli G., Cattan M. (2013). Social capital as a resource for mental well-being in older people: A systematic review. Aging Ment. Health.

[23] Downward P., Pawlowski T., Rasciute S. (2014). Does Associational Behavior Raise Social Capital? A Cross-Country Analysis of trust. East. Econ. J..

[24] Le Z., Liang H. (2020). The Impact of Social Capital on Individual Health of the Rural Elderly. J. South China Agric. Univ..

[25] Fiorillo D., Lubrano Lavadera G., Nappo N. (2020). Structural social capital and mental health: A panel study. Appl. Econ..

[26] Xue X. (2015). Social capital and national health policy. Public Financ. Res..

[27] Ehsan A., Klaas H.S., Bastianen A., Spini D. (2019). Social capital and health: A systematic review of systematic reviews. SSM Popul. Health.

[28] Gero K., Kondo K., Kondo N., Shirai K., Kawachi I. (2017). Associations of relative deprivation and income rank with depressive symptoms among older adults in Japan. Soc. Sci. Med..

[29] Smith-Frigerio S. (2020). Coping, Community and Fighting Stereotypes: An Exploration of Multidimensional Social Capital in Personal Blogs Discussing Mental Illness. Health Commun..

[30] Kilian R., Müller-Stierlin A., Lamp N., Von Gottberg C., Becker T. (2021). Criminal victimization, cognitive social capital and mental health in an urban region in Germany: A path analysis. Soc. Psychiatry Psychiatr. Epidemiol..

[31] Morgan A., Svedberg P., Nyholm M., Nygren J. (2021). Advancing knowledge on social capital for young people’s mental health. Health Promot. Int..

[32] Duren R., Yalçın Ö. (2020). Social capital and mental health problems among Syrian refugee adolescents: The mediating roles of perceived social support and post-traumatic symptoms. Int. J. Soc. Psychiatry.

[33] Delaruelle K., Walsh S.D., Dierckens M., Deforche B., Kern M.R., Currie C., Maldonado C.M., Cosma A., Stevens G.W.J.M. (2021). Mental Health in Adolescents with a Migration Background in 29 European Countries: The Buffering Role of Social Capital. J. Youth Adolesc..

[34] Bamford J., Klabbers G., Curran E., Rosato M., Leavey G. (2021). Social Capital and Mental Health Among Black and Minority Ethnic Groups in the UK. J. Immigr. Minority Health.

[35] Fan L., Zhang J., Chen J., Fei G. (2021). The influence of cultural adaptation on mental health of villagers in Traditional Ethnic Villages: A cross period study based on three Dong villages in Southeast Guizhou. J. Southwest Univ. Natl..

[36] Di Giorgi E., Michielin P., Michielin D. (2020). Perception of climate change, loss of social capital and mental health in two groups of migrants from African countries. Ann. Ist. Super. Sanita.

[37] Zhang M., Lu H., Yang L. (2021). The influence of social capital on migrant workers′ mental health—Evidence from samples in Fujian. J. Fujian Agric. For. Univ. (Philos. Soc. Sci.).

[38] Fang H. (2020). An Empirical Study of the Influence of Social Capital on the Health of Urban and Rural Elderly—Based on CGSS Mixed Section Data. J. Huazhong Agric. Univ. (Soc. Sci. Ed.).

[39] Lin N., Wang X., Peng C., Hu J., Yi K. (2021). Ruan, Z. Study on social capital and mental health of the elderly in urban and rural areas of Zhejiang province. Psychol. Mon..

[40] Perazzo J.D., Currie J., Horvat Davey C., Lambert J., Webel A.R. (2020). Depression and social capital in people living with HIV. J. Psychiatr. Ment. Health Nurs..

[41] Cao W., Li L., Zhou X., Zhou C. (2015). Social capital and depression: Evidence from urban elderly in China. Aging Ment. Health.

[42] Lu N., Jiang N., Sun Q., Lou V.W.Q. (2020). Community Social Capital and Positive Caregiving Experiences Among Adult-Children Caregivers of Older Adults with Disabilities in Urban China. Res. Aging.

[43] Downward P., Rasciute S., Kumar H. (2020). The effect of health on social capital; a longitudinal observation study of the, UK. BMC Public Health.

[44] Somefun O.D., Simo Fotso A. (2020). The effect of family and neighbourhood social capital on youth mental health in South Africa. J. Adolesc..

[45] Radloff L.S. (1977). The CES-D Scale: A Self-Report Depression Scale for Research in the General Population. Appl. Psychol. Meas..

[46] Turvey C.L., Wallace R.B., Herzog R. (1999). A Revised CES-D Measure of Depressive Symptoms and a DSM-Based Measure of Major Depressive Episodes in the Elderly. Int. Psychogeriatr..

[47] Xi C. (2019). Does Mental Health Affect Households’ participation in Risky Financial Market? Evidence from China Family Panel Studies (CFPS). Econ. Issues China.

[48] Desai R.A., Dausey D.J., Rosenheck R.A. (2005). Mental health service delivery and suicide risk: The role of individual and facility factors. Am. J. Psychiatry.

[49] Kelly B.D., Davoren M., Mhaoláin Á.N., Breen E.G., Casey P. (2009). Social capital and suicide in 11 European countries: An ecological analysis. Soc. Psychiatry Psychiatr. Epidemiol..

[50] Zhao J., Lu M. (2010). The Contribution of Guanxi to Income Inequality in Rural China and a Cross-regional Comparison: A Regression-based Decomposition. China Econ..

[51] Hang B. (2015). Gift Spending and Urban Household Consumption—An Empirical Study Based on Status Seeking. Stat. Res..

[52] Lebenbaum M., Laporte A., Oliveira C.D. (2021). The effect of mental health on social capital: An instrumental variable analysis. Soc. Sci. Med..

[53] Angrist J.D., Imbens G.W., Rubin D.B. (1996). Identification of causal effects using instrumental variables. J. Am. Stat. Assoc..

[54] Mcpherson A.C. (2016). Collaborative Community Prevention: An Ecological Approach to Mental Health Support for Children in Rural America. PhD. Thesis.

[55] Fieldhouse J. (2012). Community participation and recovery for mental health service users: An action research inquiry. Br. J. Occup. Ther..

[56] Erdem Ö., Prins R.G., Voorham T.A., van Lenthe F.J., Burdorf A. (2015). Structural neighbourhood conditions, social cohesion and psychological distress in The Netherlands. Eur. J. Public Health.

[57] Machio P.M., Kariuki P.C., Ng’ang’a A.M., Njoroge M.M. (2020). Social Capital and Women’s Empowerment in Kenya: Case Study of Murang’a County.

[58] Chen H., Zhang Y. (2019). The Impact of Pension Insurance on Fertility Desire—An Empirical Analysis Based on China General Social Survey. Insur. Stud..

[59] Chen Q. (2015). Econometrics and Stata Application.

[60] Wooldridge J.M. (2003). Introduction to Econometrics: Modern Perspective.

[61] Lian Y., Liao J. (2017). How to test the coefficient difference between groups after grouping regression?. J. Zhengzhou Inst. Aeronaut. Ind. Manag..

[62] Arezzo M.F., Giudici C. (2017). The Effect of Social Capital on Health Among European Older Adults: An Instrumental Variable Approach. Soc. Indic. Res..

[63] Reichenheim M.E., Coutinho E.S. (2011). Measures and models for causal inference in cross-sectional studies: Arguments for the appropriateness of the prevalence odds ratio and related logistic regression. J. Epidemiol. Community Health.

